# Immunotherapy and Immunochemotherapy in Visceral Leishmaniasis: Promising Treatments for this Neglected Disease

**DOI:** 10.3389/fimmu.2014.00272

**Published:** 2014-06-13

**Authors:** Bruno Mendes Roatt, Rodrigo Dian de Oliveira Aguiar-Soares, Wendel Coura-Vital, Henrique Gama Ker, Nádia das Dores Moreira, Juliana Vitoriano-Souza, Rodolfo Cordeiro Giunchetti, Cláudia Martins Carneiro, Alexandre Barbosa Reis

**Affiliations:** ^1^Laboratório de Imunopatologia, Núcleo de Pesquisas em Ciências Biológicas, Universidade Federal de Ouro Preto, Ouro Preto, Brazil; ^2^Laboratório de Pesquisas Clínicas, Ciências Farmacêuticas, Escola de Farmácia, Universidade Federal de Ouro Preto, Ouro Preto, Brazil; ^3^Instituto Nacional de Ciência e Tecnologia em Doenças Tropicais, Belo Horizonte, Brazil; ^4^Laboratório de Biologia das Interações Celulares, Departamento de Morfologia, Universidade Federal de Minas Gerais, Belo Horizonte, Brazil

**Keywords:** visceral leishmaniasis, immunology, immunotherapy, immunochemotherapy, *Leishmania infantum*, *Leishmania donovani*

## Abstract

Leishmaniasis has several clinical forms: self-healing or chronic cutaneous leishmaniasis or post-kala-azar dermal leishmaniasis; mucosal leishmaniasis; visceral leishmaniasis (VL), which is fatal if left untreated. The epidemiology and clinical features of VL vary greatly due to the interaction of multiple factors including parasite strains, vectors, host genetics, and the environment. Human immunodeficiency virus infection augments the severity of VL increasing the risk of developing active disease by 100–2320 times. An effective vaccine for humans is not yet available. Resistance to chemotherapy is a growing problem in many regions, and the costs associated with drug identification and development, make commercial production for leishmaniasis, unattractive. The toxicity of currently drugs, their long treatment course, and limited efficacy are significant concerns. For cutaneous disease, many studies have shown promising results with immunotherapy/immunochemotherapy, aimed to modulate and activate the immune response to obtain a therapeutic cure. Nowadays, the focus of many groups centers on treating canine VL by using vaccines and immunomodulators with or without chemotherapy. In human disease, the use of cytokines like interferon-γ associated with pentavalent antimonials demonstrated promising results in patients that did not respond to conventional treatment. In mice, immunomodulation based on monoclonal antibodies to remove endogenous immunosuppressive cytokines (interleukin-10) or block their receptors, antigen-pulsed syngeneic dendritic cells, or biological products like Pam3Cys (TLR ligand) has already been shown as a prospective treatment of the disease. This review addresses VL treatment, particularly immunotherapy and/or immunochemotherapy as an alternative to conventional drug treatment in experimental models, canine VL, and human disease.

## Introduction of Visceral Leishmaniasis: Epidemiology of a Zoonotic and Anthroponotic Neglected Disease

Visceral leishmaniasis (VL) is a severe chronic systemic disease caused by *Leishmania donovani* or *L. infantum*. Occasionally, *L. tropica* in the Middle East and *L. amazonensis* in South America can produce VL (1). *Leishmania* spp. are transmitted to human and animal hosts through the bite of female sand flies from the genera *Phlebotomus* in the Old World and *Lutzomyia* in the New World (2). Depending on whether or not a reservoir host is present, there are two basic types of epidemiological cycles: zoonotic, generally caused by *L. infantum*, which occurs in the Mediterranean Basin, China, the Middle East, and South America, and anthroponotic, generally caused by *L. donovani*, which is prevalent in East Africa, Bangladesh, India, and Nepal (3). The dogs, independent of the clinical form of VL, are the main urban reservoirs of *L. infantum* and represent the major source of contagion for the vectors by virtue of the high prevalence of infection and intense cutaneous parasitism (4, 5).

Canine visceral leishmaniasis (CVL) is present in approximately 50 countries, mainly in South America, the Mediterranean region, and Africa (6, 7). Several reports have revealed the emergence of canine infection in new locations, such as the United States and Canada (8, 9), and a northward spread in Europe, as found in Italy (10, 11). The seroprevalence of CVL ranges between 2 and 25% in endemic areas of Europe (2) and 5.9 and 29.8% in Brazil (12). In recent years, with the development of molecular techniques, infection rates have been shown to be underestimated. Studies in Europe have demonstrated an elevated prevalence of CVL (60–80%) by polymerase chain reaction (PCR) compared with serology (25%) (13). During a cross-sectional study in an urban area of Brazil, we observed that approximately a quarter of seronegative dogs were infected by *L. infantum* according to PCR (14), and they had approximately twice the risk of seroconversion as those that were PCR negative (15). Finally, a high incidence of infection was demonstrated by PCR in endemic areas (16).

Official global estimates indicate that there are more than 58,000 cases of human VL (HVL) per year. However, the number may actually be as high as 0.2–0.4 million, and more than 90% of cases occur in five countries: India, Bangladesh, Sudan, Brazil, and Ethiopia (17). The incidence of VL is relatively low in southern Europe (2), but the disease has recently spread further northward as shown by reports of cases in northern Italy (18) and Germany (19). Additionally, the epidemiology of the disease has been influenced by the expansion of human immunodeficiency virus (HIV). Of the 70 countries that are endemic for VL, 35 have reported cases of *Leishmania*–HIV co-infection (20). One of the critical complications associated with co-infection is that HIV reduces the likelihood of a therapeutic response to treatment against *L. infantum*, and it also greatly increases the probability of a relapse (21).

Visceral leishmaniasis is clinically characterized by prolonged fever, weakness, anorexia, weight loss, hepatomegaly, splenomegaly, hypergammaglobulinemia, and pancytopenia. Without treatment, the disease may progress over time to severe cachexia, multisystem disease, bleeding, secondary infections, and death (22, 23). The case-fatality rates range from 1.5% in Bangladesh to 2.4% in India and 6.2% in Nepal (17). However, studies conducted by Ahluwalia et al. (24) in Bangladesh and by Barnett et al. (25) in India suggest that the rates are probably underestimated. In Brazil, data from the Ministry of Health were used to estimate 6.5% mortality from 2001 to 2011 (26). VL results in death mainly in untreated patients. The majority of leishmaniasis deaths go unrecognized, and even with treatment access, case-fatality rates can be as high as 10–20% (17). These findings underscore the need for further studies on the development of immunotherapeutic and prophylactic strategies for VL and *Leishmania*–HIV co-infection.

In this review, we discuss the recent advances in immunotherapy and immunochemotherapy in the treatment of VL, focusing on both canine and human disease and experimental models (murine). We also discuss some aspects of the epidemiology and immunology of VL, the most recent strategies and guidelines for chemotherapy, and new advances in modulating the host immune response (collectively called immunotherapy) with or without conventional chemotherapy.

## Immunobiology of Visceral Leishmaniasis: Cells and Immune Mediators Related to Resistance and Susceptibility

In visceral disease, the immunology and immunopathology in humans, dogs, and experimental rodent models has been extensively studied, with many points characterized and others still to be elucidated (27–29). A general consensus is that despite the peculiarities of each model, the outcome of the disease is critically influenced by the host immune response.

Several studies have demonstrated that susceptibility to HVL is related to a high titer of circulating antibodies and a depression of type-1 T cell-mediated immunity, mainly with decreased production of interferon (IFN)-γ and interleukin (IL)-12, including a marked up-regulation of IL-4 and IL-10 cytokines (30–32). In CVL, the protective response has also been associated with activation of Th1 cells producing IFN-γ, IL-2, and tumor necrosis factor (TNF)-α (33, 34). Similar to HVL, active CVL is characterized by polyclonal B-cell activation, specific immunosuppression, and the appearance of clinical symptoms depending on the parasite density in different visceral organs (35, 36). An interplay of Th1 and Th2 cytokines appears to exist during *Leishmania* infection, and this suggests important roles for different cytokines in disease protection and pathogenesis (37).

The innate immune response contributes to VL resistance, acting to control parasite growth during the early stages of infection. Furthermore, it directs cell recruitment and helps develop the cytokine microenvironment in which parasite-specific T cells are primed (38, 39).

The control of VL infection depends on a successful cell-mediated immune response (40), in which IFN-γ, produced mainly by CD4^+^ T cells and natural killer (NK) cells stimulated by IL-12, leads to stimulation of microbicide action mediated by nitric oxide (41, 42). TNF-α exerts cytotoxic effects on invading parasites via its receptor, TNFR (43). There have been reports of the involvement of different Th17 cytokines in HVL, including IL-17, IL-22 (44), and IL-21 (45), which are important in the migration, recruitment, and activation of neutrophils. Recent work of Gautam et al. (46) evaluating patients with VL showed that individuals with active disease exhibit predominantly anergic splenic CD8 cells and CD8 peripheral blood mononuclear cells (PBMCs) with a mixture of anergic cells and cytotoxic T lymphocytes (CTLs). Following a cure after treatment, CD8 T cells contribute to *Leishmania*-induced IFN-γ production. The authors suggested that CD8 T cells are driven to anergy/exhaustion in HVL, which affects their ability to launch a protective immune response (46).

The expression of the various chemokine genes is observed in *Leishmania* infection (47, 48). Chemokines have been shown to play a crucial role in determining the outcome of leishmaniasis by coordinating the leukocyte recruitment involved in innate and adaptive immune responses (49, 50). Patients with VL show elevated concentrations of CXCL9 and CXCL10 in their serum during active infection, and it has been suggested that these chemokines play an important role along with IFN-γ in the disease (51). Dogs naturally or experimentally infected with *L. infantum* have CXCL10 mRNA overexpressed in the spleen, leading to a substantial type-1 immune response (52). A detailed analysis of chemokine expression in skin samples from dogs naturally infected with *L. infantum* demonstrated enhanced parasite density and a positive correlation with CCL2, CCL4, CCL5, CCL21, and CXCL8 (49). It is noteworthy that some chemokines such as CCL2 can activate macrophages to participate in reducing the parasite load (53).

The monocytes/macrophages, the main targets of *Leishmania*, represent one of the first steps of the innate immune response to kill intracellular parasite (54). The survival of the parasite relies on evasion mechanisms such as the modulation of leishmanicidal activity of macrophages by production of tumor growth factor (TGF)-β with deactivation, inhibition of the action of IFN-γ, reduced expression of MHC class II molecules, and suppression of nitric oxide production (55). IL-10 is another cytokine produced by macrophages that contributes to the survival of *Leishmania* in these cells, and it has emerged as the most potent factor for VL pathogenesis. It inhibits synthesis of cytokines produced by macrophages, such as IL-1β, IL-6, IL-8, and TNF-α (56) and reduces the antigen-presenting function of these cells by decreasing the expression of MHC class II molecules (57). The association of IL-10 and VL in humans with active disease is well-established (32). Others cytokines, such as IL-27 and IL-21, have also emerged as being implicated in disease progression through the regulation of IL-10 (45). Other cells, such as NK cells, are important components of the immune response to combat infection. They connect the innate response to the development of efficient adaptive cellular immunity, mainly through TNF-α and IFN-γ production (58).

Successful immunity against *Leishmania* involves a complex immunological response of several mechanisms and factors, including the migration of appropriate cell populations to the infected sites, cytokine microenvironment, chemokines, and others. The elucidation and a better understanding of the immune response against *Leishmania* infection are relevant to establish a rational approach for immunomodulatory therapy and vaccine development.

## Conventional VL therapy

The drug policy in endemic countries and therapeutic decisions should be based on the individual benefit–risk ratio of drugs, the health service setting, and the availability of anti-leishmanial medicines in the context of public health concerns and the difference of the VL epidemiological aspects (anthroponotic and zoonotic) (59). For example, 70% of the anthropozoonotic VL burden occurs in the Indian subcontinent (17), and a critical challenge is related to widespread resistance to pentavalent antimony; resistance rates approach 60% in Bihar, India (60). In Europe, Asia, Africa, and the Americas, where zoonotic cases are observed, the risk of human disease is well-known to be associated with canine infection rates (61). Another serious problem that mainly occurs with zoonotic VL is that canine treatment does not effectively lead to a parasitological cure since these animals are constant sources of infection for sand flies (36).

Nevertheless, a few drugs are available. In most cases, the first-line treatment is pentavalent antimonials, and amphotericin B or pentamidine are commonly employed as second-line medicines. In recent years, other medicines have been extensively studied and became invaluable, such as liposomal amphotericin B (62), miltefosine (63, 64), and paromomycin (65). In line with this, current World Health Organization (WHO) treatment advice varies by global region, which is partially explained by differences in parasite susceptibility (59, 66, 67) (Table 1). Even so, the number of VL cases is increasing worldwide, and the enduring problems with current chemotherapy tools are still a critical issue. Furthermore, in many developing countries the cost of treatment is the greatest challenge faced by health authorities (Table 2). In the following section, we briefly review conventional chemotherapy, stressing essential issues in HVL and studies using different drugs and strategies for canine disease.

**Table 1:** **Recommendations of the World Health Organization for the treatment of visceral leishmaniasis per geographic region ranked by preference [World Health Organization (59)]**.

**ANTHROPONOTIC VISCERAL LEISHMANIASIS CAUSED BY *L. donovani* IN THE INDIAN SUBCONTINENT**
Liposomal amphotericin B: 3–5 mg/kg daily over 3–5 days to a total dose of 15 mg/kg by infusion or 10 mg/kg as a single dose
Combination therapy (co-administered following the sequence): (i) liposomal amphotericin B (5 mg/kg by infusion, single dose) + miltefosine (daily for 7 days, dosage as below), (ii) liposomal amphotericin B (5 mg/kg by infusion, single dose) + paromomycin (daily for 10 days, dosage as below), (iii), miltefosine + paromomycin both for 10 days (dosages as below)
Amphotericin B deoxycholate: 0.75–1.0 mg/kg daily or on alternate days for 15–20 doses by infusion
Miltefosine: children aged 2–11 years, 2.5 mg/kg daily; 12 years and older <25 kg body weight, 50 mg/day; 25–50 kg, 100 mg/day; >50 kg, 150 mg/day; orally for 28 days
Paromomycin: 15 mg (11 mg base)/kg/day by intramuscular route for 21 days
Pentavalent antimonials: 20 mg Sb^V^/kg/day intramuscularly or by infusion for 30 days (areas where they are effective: Bangladesh, Nepal, and the Indian states of Jharkhand, West Bengal, and Uttar Pradesh)
**VISCERAL LEISHMANIASIS CAUSED BY *L. donovani* IN EAST AFRICA**
Combination therapy: pentavalent antimonials (20 mg Sb^V^/kg/day intramuscularly or by infusion) + paromomycin [15 mg (11 mg base)/kg/day by intramuscular route] for 17 days
Pentavalent antimonials: same treatment scheme as above
Liposomal amphotericin B: 3–5 mg/kg daily given over 6–10 days for a total dose of 30 mg/kg by infusion
Amphotericin B deoxycholate: same treatment scheme as above
Miltefosine: same treatment scheme as above
**VISCERAL LEISHMANIASIS CAUSED BY *L. infantum***
Liposomal amphotericin B: 3–5 mg/kg daily over 3–6 days for a total dose of 18–21 mg/kg by infusion
Pentavalent antimonials: 20 mg Sb^V^/kg/day intramuscularly or by infusion for 28 days
Amphotericin B deoxycholate: 0.75–1.0 mg/kg daily or on alternate days for 10–20 doses by infusion (total dose: 2–3 g)

**Table 2:** Cost of visceral leishmaniasis treatment (patient weighing 35 kg)*.

Medicine (compound)	Treatment regimen in days	Drug cost in US$
L-Amb 10 mg/kg	1	125
L-Amb 20 mg/kg	2–4	250
Amphotericin B deoxycholate 1 mg/kg (alternating days)	30	20
MF 100 mg/kg	28	65–150
PM 15 mg/kg/day	21	15
SSG 20 mg/kg/day	30	55
MA 20 mg/kg/day	30	59
L-Amb 5 mg/kg + MF 100 mg/kg	8	88–109
L-Amb 5 mg/kg + PM 15 mg/kg/day	11	78
MF 100 mg/kg + PM 15 mg/kg/day	10	30–60
SSG 20 mg + PM 15 mg/kg/day	17	43

*Calculations for SSG and MF based on exchange rate of €1 = US$ 1.40 (December 2013). Price range of MF depends on order volume. Price is based on generic SSG, World Health Organization (59).

*L-Amb, liposomal amphotericin B; MF, miltefosine; PM, paromomycin; SSG, sodium stibogluconate; MA, meglumine antimoniate*.

## Pentavalent Antimonials

It is generally accepted that pentavalent antimonials (Sb^V^) are the pro-drug, and that they must convert to trivalent antimonials (Sb^III^) to have anti-leishmanial activity. The issues with the use of this drug are commonly attributed to serious side effects such as cardiotoxicity (68), pancreatitis (69), and nephrotoxicity (70). The doses and treatment durations of Sb^V^ have undergone constant changes over the years. The use of Sb^V^ in canine therapy does not lead to clinical and parasitological cure (71), and disease relapses are common (72). Moreover, prolonged or repeated use of this drug can induce resistance in *Leishmania* clones (73). Currently, an important strategy for therapy in dogs is the use of liposome-encapsulated Sb^V^, which promotes improved clinical status and reduced parasite load in infected animals (74).

## Amphotericin B Deoxycholate and Liposomal Amphotericin B

The anti-*Leishmania* activity by amphotericin B is due to its complexation with 24-substituted sterols such as ergosterol and episterol, which are predominant in the plasma membranes of parasites. Amphotericin B deoxycholate is generally used for cases that are unresponsive to Sb^V^, and it is a first-line drug in India. Unresponsiveness and relapses occur rarely and mostly in relation to HIV co-infection (75). The major limitation to using this drug is the necessity for prolonged hospitalization and close monitoring due to its high nephrotoxicity (76). The liposomal formulation improves the safety profile of amphotericin B and increases the anti-leishmanial activity, with selectivity to macrophage reticular–endothelial system (77). There are three formulations, liposomal amphotericin B, amphotericin B lipid complex, and amphotericin B cholesterol dispersion; all of which ensure a decrease in nephrotoxicity. Currently, liposomal amphotericin B is the first treatment choice for HVL in several endemic countries in Europe as well as in the United States. Following other countries, the Ministry of Health in Brazil, expanded the use of this medicine in the last years. In dogs, therapy with amphotericin B deoxycholate reduces serum antibody levels and parasite loads and increases the lymphoproliferative response, but the effects are transitory (78). In addition, renal failure is a common outcome (79), and the drug is not recommended for canine therapy. Treatment with liposomal amphotericin B resulted in recovery in dogs, but despite the initial effectiveness, relapses can occur (78, 80).

## Miltefosine

Miltefosine, which was initially developed as an anticancer drug, is the first effective oral drug for VL, and it represents a great breakthrough (81, 82). The main anti-leishmanial activity is due to modulation of cell surface receptors, inositol metabolism, phospholipase activation, and protein kinase C in addition to mitogenic pathways resulting in apoptosis (83). The main side effects of the drug include gastrointestinal disturbances, but the symptoms are transient or reversible; however, teratogenicity is a major problem (84). Careful use of this drug should be mandatory, since resistance can be easily induced in *in vitro* experiments (85). Miltefosine has recently emerged as a potential tool for CVL treatment, and its use has been evaluated in monotherapy and in combination with other drugs (86, 87). There are no nephrotoxic effects reported, and vomiting is the most common side effect in dogs (88).

## Paromomycin

Paromomycin presents variable efficacy in distinct parts of the world (89). The drug’s low-cost, relatively short duration of administration, and good safety profile strengthens its usefulness as a first-line drug (90). The drug has activity against *Leishmania* by altering plasma membrane fluidity, interfering in ribosomal function, and disrupting mitochondrial membrane potential (91). The most common side effects associated with paromomycin are ototoxicity and impaired liver function (92). Although it is the least expensive drug for VL, current demand for paromomycin is low, and production is irregular. In canine studies, the drug was associated with a decrease in anti-*Leishmania* IgG antibody titers (93). Following clinical recovery, relapse, and parasitologic cure in symptomatic CVL treated with paromomycin, only clinical improvement was verified (94). However, the search for an optimum dosage for the safe use in the treatment of CVL is necessary.

## Combined Drug Therapy

In general, the treatment of VL is clinically challenging, and the drugs have several drawbacks. Over the past few years, the WHO consensus has evolved toward the use of combination regimens, particularly in highly endemic regions. Combining drugs from various chemical classes has the following objectives: (i) shortening the duration of treatment, reducing total parenteral drug doses with fewer toxic effects, and improving adherence to the regimen; (ii) lowering the cost of the treatment (less burden on the health system), thus providing a more cost-effective option, and (iii) helping to delay the emergence of resistance. These strategies could increase the therapeutic lifespan of the respective drugs, as has been demonstrated with drugs for diseases like malaria, tuberculosis, and HIV. These strategies might also encourage a cure, especially in complicated cases like *Leishmania*–HIV co-infection, for which treatment outcomes with monotherapy have been consistently poor (1, 59, 66).

Recently, reports of treatment failure with Sb^V^ from the Indian subcontinent have increasingly raised the issue of acquired drug resistance (67). This concern also extends to miltefosine, which is worrisome given the drug’s long half-life (84). More recently (95) reported unresponsiveness to liposomal amphotericin B in Sudanese patients, who experienced cured disease only with combination treatment. Specifically, a 17-day combination of antimonials with paromomycin presented 93% efficacy in East Africa. Combination regimens including liposomal amphotericin B (single dose), paromomycin, and/or miltefosine were also found to be extremely effective (98–99%) and safe, and are now included in WHO guidelines for the Indian subcontinent (see Table 1) (1, 59).

Substantial progress has been made in the chemotherapeutic approaches in recent years, but the current conventional drugs for VL are far from ideal (96). Combined therapy enhancement should be on-going, but exploratory studies that encompass highly efficient regimens in single dose treatments are urgently needed (97). The most effective strategies for protecting against resistance are uncertain, but overall monitoring of access to anti-leishmanial drugs should definitely be strengthened. In this context, canine treatment is still controversial, and strict action should be taken particularly for zoonotic VL. Worryingly, in Europe, dogs with active VL are routinely treated with first-line drugs for HVL, and this practice could generate parasites that are resistant to conventional therapies (98). Considering the success of combined therapy, the control and the effectiveness of current conventional medicines must be protected until new options arise.

## Promising Strategies for VL Treatment: Immunotherapy and Immunochemotherapy

The immunotherapy, involves the use of biological substances or molecules to modulate the immune responses for the purpose of achieving a prophylactic and/or therapeutic success. Currently, immunotherapy is a strategy applied against various diseases such as cancer, allergies, and some viruses (hepatitis). It is based on the idea that our organism’s defense systems are capable of protecting us against a variety of diseases (in most circumstances). Normally, it is known that disease occurs when there is either a failure, suboptimal, or excessive immune response and this could be remedied by appropriate immune modulation or interventions using immunomodulatory agents or biological response modifiers. Thus, immunotherapeutic agents can exert their effect by directly or indirectly augmenting the host natural defenses, restoring the impaired effectors functions or decreasing host excessive response (99–101). Moreover, the combination of immunotherapy with chemotherapeutic drugs (immunochemotherapy), especially when applied against infectious diseases, results in an increased synergic action with activation of the immune system and direct action of drugs against the infectious agent. Therefore, immunotherapy and immunochemotherapy have been used to accelerate the specific immunity in responsive and non-responsive patients (102, 103). The underlying idea is to selectively induce Th1 responses that are fundamental for resistance in VL. Protective immunity usually follows recovery from leishmaniasis in immunocompetent patients, but the behavior of disease in these individuals suggests that their immune responses are not sterile. VL has emerged as an important opportunistic infection associated with HIV, with the risk of developing active/severe disease increasing 100–2320 times the average (20). Depending on the stage of infection and the clinical condition, the use of conventional chemotherapy can be inefficient. In such cases, combination therapy with immunomodulators that potentiate the cellular immune response can lead to more satisfactory results.

Immunotherapy with or without chemotherapy has been used for the treatment of cutaneous leishmaniasis (CL) in the last two decades. Convit et al. (104), using three injections of a vaccine composed of a lysate of *L. mexicana amazonensis* with BCG as an adjuvant, demonstrated a 94% of cure rates in CL patients in Venezuela. These authors also showed that 5341 patients from four different regions of Venezuela, who had different forms of CL (mucosal and chronic CL) and received the vaccine treatment between 1990 and 1999, demonstrated a high cure rate (91.2–98.7%) (105). In Brazil, Mayrink et al. (106) evaluated an immunotherapy protocol using a mixture of five strains of *Leishmania* vaccine and observed a 76% cure rate in patients with CL. Moreover, years later, Mayrink et al. (107) used repeated daily doses of killed *L. amazonensis* in a human clinical trial comprising 542 patients and observed that 98.1% of the individuals treated with immunotherapy (*n* = 53; *L. amazonensis* vaccine + BCG) showed a clinical cure. A similar cure rate was found in patients treated with conventional chemotherapy and an immunochemotherapy scheme (100%). The immunochemotherapy protocol was also associated with a reduction in the total volume of the drug used (17.9%) and a shorter treatment time (94.6 days for chemotherapy alone to 64.7 days for immunochemotherapy) (107). In the Sudan, a trial involving patients with persistent post-kala-azar dermal leishmaniasis and using a vaccine composed of a mixture of killed *L. major* adsorbed on alum + BCG, given four times at weekly intervals, showed that the cure rate with immunochemotherapy was significantly higher than with chemotherapy alone (final cure rates: 87 and 53%, respectively) (108).

As we observed, therapeutic vaccines in CL can be rapidly evaluated at lower cost, appear to be safe, and are not associated with the adverse effects of conventional treatment, encouraging the use of this strategy for treatment of VL. Furthermore, using immunomodulators to enhance host immunity combined with conventional chemotherapy may have several advantages as a means to improve current therapeutic regimens in this neglected disease (109). On this topic, we discuss advances in immunotherapy and immunochemotherapy for VL by focusing mainly on approaches used in humans and dogs (Table 3) and recent advances in murine models.

**Table 3:** **Immunotherapy and immunochemotherapy strategies against VL for humans and dogs**.

Immunotherapeutic agent	Chemotherapy agent	Visceral disease	Improvements	Treatment efficacy	Reference
IFN-γ	Sb^V^	Human	Accelerated parasitological control, enhanced the clinical efficacy of conventional Sb^V^ treatment, 83.2% cure rate	Marked	(109–111)
IFN-γ for 15 or 30 days (10^7^ U/mg/day)	Sb^V^ (20 mg/kg/day) at 30 days	Human	No difference was observed in patients treated with Sb^V^ alone	Moderate	(112)
IFN-γ	Sb^V^ (20 mg/kg/day) at 30 days	Human	All patients responded clinically to treatment, more quickly splenic culture-negative	Moderate	(113)
Antigenic preparation of *L. infantum* (soluble antigen)	100 mg/kg SC of *N*-methyl-d-glucamine antimoniate	Canine	Increase in the T lymphocytes, especially CD4/TcRαβ^+^ and CD4/CD45RA^+^ cells in PBMC; reduction of infection to *Phlebotomus perniciosus*	Low	(114)
Enriched-Leishmune^®^ vaccine (plus 0.5 mg of saponin)	n.d.	Canine	Higher levels of anti-FML IgG (IgG2), positive delayed type hypersensitivity reaction, lower clinical scores	Moderate	(115, 116)
Enriched-Leishmune^®^ vaccine (plus 0.5 mg of saponin)	Allopurinol (10 mg/kg) and amphotericin B (0.5 mg/kg)	Canine	Positive DTH reaction, reduction of symptomatic cases and low numbers of animals with parasites in lymph nodes and deaths	Marked	(117)
Vaccine composed by 20 μg of rLeish-110f^®^ + 25 μg of MPL-SE^®^	100 mg/kg/day IM of Glucantime^®^	Canine	Improvement in the clinical parameters (hematological, biochemical, cellular); reduction in parasitological positive animals (bone marrow smears or culture); reduced number of deaths; 33% xenodiagnosis negative of by PCR	Marked	(118)
Vaccine composed by 20 μg of Leish-111f^®^ plus 20 μg of MPL-SE^®^	20 mg/kg/day IV of Glucantime^®^	Canine	Cure rates 50%; 92% clinical improvement	Moderate	(119)
Immunomodulator P-MAPA (2.0 mg/kg) intramuscularly	n.d.	Canine	Increase CD8^+^ T cells, IL-2, and IFN-γ; decrease in IL-10 and improvement in clinical signs and reduction in parasite load in skin	Marked	(120)

*SC, subcutaneous; IM, intramuscular; IV, intravenous; n.d., not done*.

## Approaches Used in Humans

Increasing reports of treatment failure (Sb^V^, miltefosine, and liposomal amphotericin B) and complicated cases (*Leishmania*–HIV co-infection) in HVL increase the urgency of using combination therapies and developing new treatment strategies for the disease (67, 95). In fact, the added effects produced by immunotherapy and/or immunochemotherapy could be potentially useful against HVL; however, these approaches are still very rarely used.

In this context, IFN-γ is well-recognized as a cytokine capable of inducing macrophages to kill intracellular *Leishmania* (110). It is clinically well-tolerated (111), and repeat treatment with IFN-γ plus Sb^V^ has been shown to be effective in patients with Sb^V^-refractive disease, yielding a >80% cure rate in VL (112, 113, 121). Studies in untreated patients with VL demonstrated that the addition of IFN-γ as immunotherapy accelerated parasitological control (122, 123) and enhanced the clinical efficacy of conventional Sb^V^ therapy (123). However, another human trial in India showed no differences among patients treated with Sb^V^ alone (30 days, 20 mg/kg/day), Sb^V^ plus IFN-γ (30 days, 10^7^ U/mg/day), or Sb^V^ plus IFN-γ for 15 days (114). Six months after treatment, a low percentage of individuals were cured (36, 49, 42%, respectively), but the immunochemotherapy protocol was the most efficient.

A similar study was conducted in Kenyan patients with VL treated for 30 days with either conventional therapy with Sb^V^ or immunochemotherapy (daily Sb^V^ plus IFN-γ) (122). All patients responded clinically to treatment, and microscopic splenic aspirate scores rapidly decreased in both groups. Interestingly, the patients treated with immunochemotherapy had a negative spleen culture more quickly, which may demonstrate the potential of this protocol to accelerate early parasitological control (122). These results suggest the beneficial effects of using IFN-γ in the treatment of HVL. The combination of this immunotherapy or another (therapeutic vaccines, immunomodulators) with other drugs (miltefosine, liposomal amphotericin B) could provide more satisfactory results with better cure rates mainly in VL patients unresponsive to Sb^V^.

## Progress for VL Treatment in Dogs

The drugs generally used to treat CVL are highly toxic, expensive, and ineffective. They promote clinical remission without parasite reduction or sterilization, and once the treatment is withdrawn, relapses of the disease are always observed (115). Moreover, the WHO does not recommend the use of human chemotherapy in dogs due to concerns about selecting for drug-resistant parasites, which might then be untreatable in subsequent HVL infection. Also, primary resistance to these drugs is considerable, and treated dogs still have parasites in different organs even if they are asymptomatic (116).

Along with vaccine development, new drugs and new treatment strategies (immunotherapy and immunochemotherapy) are the most important alternatives for CVL control. Guarga et al. (117) evaluated the efficacy of a novel immunochemotherapy protocol in dogs naturally infected with *L. infantum*. The protocol consisted of 21 consecutive subcutaneous injections of *N*-methyl-d-glucamine antimoniate (100 mg/kg) and three applications of an antigenic preparation of *L. infantum* (soluble antigen). The animals showed an increase in the proportion of T lymphocytes, especially of CD4/TcRαβ^+^ and CD4/CD45RA^+^ cells in PBMCs, and reduction in the infection from *Phlebotomus perniciosus* after immunochemotherapy (117).

Different studies are being done to evaluate the potential of fucose–manose-ligand (FML) antigen plus saponin as an immunotherapy. Borja-Cabrera et al. (118) used three vaccine doses (1.5 mg FML +1 mg saponin) in asymptomatic dogs and observed them for 22 months after immunotherapy was complete. No deaths due to disease were recorded, and 90% of the dogs remained asymptomatic, healthy, and parasite free. In contrast, 37% of kala-azar deaths were recorded in non-treated animals (118). Another vaccine formulation (enriched-Leishmune^®^ vaccine plus 0.5 mg of saponin) was evaluated by Santos et al. (119) in dogs experimentally infected with *L. infantum*. The enriched-Leishmune was injected when dogs were seropositive and symptomatic. After immunotherapy, the dogs showed higher levels of anti-FML IgG (higher IgG2 and lower IgG1), positive delayed type hypersensitivity reactions, lower clinical scores, and normal CD4^+^ counts (119). The association of enriched-Leishmune vaccine with chemotherapy (allopurinol or amphotericin B/allopurinol) demonstrated synergistic efficacy in naturally infected animals. For both immunotherapy and immunochemotherapy, dogs showed an intradermal response to *Leishmania* antigen, reduction of symptomatic cases, a lower proportion of animals presenting with parasites in lymph nodes, and fewer deaths (120).

Miret et al. (124) evaluated immunochemotherapy using Leish-110f^®^ + MPL-SE^®^ vaccine in combination with Glucantime^®^ and showed in symptomatic dogs improved clinical parameters (hematological, biochemical, and immunological), reduced parasite-positive animals, and reduced number of deaths compared to control groups (adjuvant alone or placebo). Trigo et al. (125) performed two separate trials to evaluate the recombinant polyprotein vaccine antigen Leish-111f^®^, formulated with MPL-SE^®^ for therapeutic purposes against CVL. In both trials, a therapeutic efficacy of the vaccine in preventing mild cases of disease was demonstrated, and weekly injections (three doses) promoted clinical cure for many dogs with VL.

Using an immunomodulator, Santiago et al. (126) tested the immunotherapeutic effect for CVL of a protein aggregate of magnesium–ammonium phospholinoleate–palmitoleate anhydride (P-MAPA) obtained by fermenting the fungus *Aspergillus oryzae*. P-MAPA showed immunomodulatory activity, with greater stimulation of cellular immunity and no toxic effects in mice and dogs (127). To investigate the immunotherapeutic potential of P-MAPA, symptomatic dogs were submitted to a protocol of 15 doses of the immunomodulator (2.0 mg/kg) intramuscularly. An increase in CD8^+^ T cells in peripheral blood, a decrease in IL-10 levels, and an increase in IL-2 and IFN-γ, improved clinical signs, and reduced skin parasitism were obtained after immunotherapy (126).

Some CVL vaccines candidates have been developed by our research group, called LBSap and LBSapSal, demonstrating important results of immunogenicity and efficacy in phase I and II trials (128, 129). Currently, we are investigating the potential immunotherapeutic of these and other vaccines in the treatment of CVL. Given these results, we believe that we could use immunotherapy/immunochemotherapy to treat dogs in endemic areas to eliminate their reservoir condition mainly by decreasing the skin parasite load, which would block the zoonotic transmission cycle.

## Recents Advances in Murine Models

With the current status of *Leishmania* treatment, use of a low-dose drug or a short course of an effective drug in combination with an immunomodulator is an approach for effective treatment of disease (130). Thus, murine models of leishmaniasis are being extensively used to obtain preliminary information on the anti-*Leishmania* potential of different compounds (67). Many researchers have worked on the development and discovery of new agents against the parasite, and several studies have shown that the use of immunotherapy would be an important tool in control of VL.

Because Sb^V^-based anti-leishmanial chemotherapy depends in part on the Th1 response, which can be induced by dendritic cell (DC)-based treatment (131). DC-based immunotherapy combined with Sb^V^ chemotherapy was very effective against murine VL (132). While three weekly injections of *L. donovani*-soluble, antigen-pulsed syngeneic bone marrow-derived DCs into mice infected with *L. donovani* only reduced the number of spleen and liver amastigotes, when combined with sodium stibogluconate, the treatment resulted in a complete eradication of the parasites from both organs (132).

A fusion protein that stimulates T cells through OX40, as well as a monoclonal antibody (mAb) agonist against CD40, enhanced host immunity, and supported low-dose Sb^V^ in a murine VL model (133, 134). The treatment enhanced both the rate of granuloma maturation and CD4^+^ T cell proliferation and promoted greater reduction in the parasite burden, without causing excess tissue damage. Moreover, the blockade of cytotoxic T lymphocyte-associated (CTLA)-4, a negative regulator of T cell co-stimulation using mAb, has a beneficial effect in experimental VL, inducing the destruction of 60% of the parasites within liver macrophages, stimulating IFN-γ secretion, and enhancing mononuclear cell recruitment with significant synergy with Sb^V^ (134).

In VL, cytokine-mediated immunosuppression is dominated by IL-10 and TGF-β (135). Hence IL-10-deficient mice are highly resistant to VL (27, 135). This cytokine also impairs responsiveness to Sb^V^. In experimental models of VL, treatment with mAb against the IL-10 receptor allowed a 10-fold reduction in the effective dose of Sb^V^ compared with the drug alone, as well as considerable shortening of the time needed for effective therapy (135, 136). Inhibition of TGF-β has been shown to decrease parasite burdens in experimental VL; however, TGF-β blockade has no apparent effect on Sb^V^ activity (136).

Using lower doses of miltefosine in combination with Pam3Cys (an immunomodulator synthetic bacterial lipopeptide (BLP) and TLR-2/1 ligand) in a BALB/c mouse model of VL, Shakya et al. (137) demonstrated significantly enhanced parasitic inhibition and Th1 cytokine production and an increased phagocytosis index. Another study, conducted by Karmakar et al. (138), demonstrated the interactions between a TLR ligand and invariant natural killer T (iNKT) cell activation as immunotherapy in VL. The authors evaluated the anti-*Leishmania* immune responses and the protective efficacy of the b-(1–4)-galactose terminal NKT cell ligand glycosphingophospholipid (GSPL) antigen of *L. donovani* parasites. Their findings suggested that TLR4 can function as an upstream sensor for GSPL and promote intracellular inflammatory signaling necessary for parasite killing. Furthermore, the treatment with GSPL induced a strong, effective T cell response, with control of acute parasite burden leading to undetectable parasite persistence (138).

The remarkable improvement in clinical signs and decrease in parasite burden in the immunotherapy or immunochemotherapy protocols described mostly arise from the restoration and activation of an effective immune response. In this context, the search for new therapeutic vaccines or substances with strong immunomodulatory effects as adjuvants (immunotherapy) may lead to the next generation of drugs, and associations with conventional chemotherapy (immunochemotherapy) will form the treatment strategy to cure visceral disease or reverse severe clinical forms of HVL.

## Concluding Remarks

Most traditional and low-cost treatment options for VL are toxic and have many side effects, and the use of more effective drugs is limited mainly by the high cost. Successful immunotherapy using killed parasite vaccines or immunomodulators has been extensively reported in leishmaniasis. Another approach is immunochemotherapy, in which a low-dose or short course of chemotherapy associated with a vaccine or immunomodulator quickly induces an effective immune response. In VL, many efforts in the development and application of immunotherapy or immunochemotherapy have been made in the last decade, mainly due to the emergence of drug resistance and the increase in HIV co-infection. Many researchers have treated CVL using vaccines and immunomodulators with or without chemotherapy. In humans, the use of cytokines like IFN-γ associated with Sb^V^ has demonstrated promising results in patients that are unresponsive to conventional treatment. In murine models, immunomodulation based on mAbs to remove endogenous immunosuppressive cytokines or block their receptors, antigen-pulsed syngeneic DCs, and biological products like Pam3Cys (TLR ligand) has demonstrated future prospects for the treatment of VL. Efforts need to be directed to standardization and additional carefully controlled studies in animals and humans to understand the immunologic basis of these new vaccines and other immunomodulators in conjunction with chemotherapeutic agents for treatment of this important neglected disease.

## Conflict of Interest Statement

The authors declare that the research was conducted in the absence of any commercial or financial relationships that could be construed as a potential conflict of interest.
